# Sympathetic Ophthalmia as a Complication of Untreated Choroidal Melanoma

**DOI:** 10.3390/jcm14155579

**Published:** 2025-08-07

**Authors:** Tomasz Chorągiewicz, Paweł Oszczędłowski, Joanna Dolar-Szczasny, Mario Damiano Toro, Olga Denysiuk, Adam Słoka, Alicja Chorągiewicz, Yacoub A. Yousef, Robert Rejdak

**Affiliations:** 1 Chair and Department of General and Pediatric Ophthalmology, Medical University of Lublin, 20-079 Lublin, Poland; 2 Eye Clinic, Public Health Department, Federico II University, Via Pansini 5, 80131 Naples, Italy; 3 Medical Faculty, Shupyk National Healthcare University of Ukraine, 04112 Kyiv, Ukraine; 4 Provincial Eye Hospital in Krakow, 31-723 Krakow, Poland; 5 Faculty of Medicine, Medical University of Bialystok, 15-222 Bialystok, Poland; 6 Department of Surgery (Ophthalmology), King Hussein Cancer Centre, Amman 11941, Jordan

**Keywords:** sympathetic ophthalmia, uveal melanoma, choroidal melanoma

## Abstract

We present a rare case of sympathetic ophthalmia in the fellow eye of a 59-year-old Caucasian male diagnosed with untreated malignant choroidal melanoma. Initially identified with a medium-sized choroidal melanoma, the patient declined recommended brachytherapy and did not seek medical attention for two years. Upon returning, he exhibited signs of sympathetic ophthalmia in the contralateral eye. Treatment with corticosteroid-based immunosuppression was initiated. After consenting to treatment for the melanoma, the affected left eye was enucleated, and histopathology confirmed spindle cell choroidal melanoma. This case underscores the uncommon development of sympathetic ophthalmia without prior ocular trauma or surgery, linked to untreated choroidal melanoma.

## 1. Introduction

Sympathetic ophthalmia (SO) is a rare, diffuse granulomatous, bilateral panuveitis with an incidence of 33 per 100,000 persons with open globe injury/years [1]. The most common cause of this disease is an ocular injury, with the recent (2022) meta-analysis estimating that SO is a complication of 0.19% of ocular injuries [1]. The eye is an immune-privileged organ in which systemic immune response is reduced in physiological conditions [2]. Pathogenesis of SO is linked to the disruption of the blood–retina barrier, an event in which previously unexposed ocular antigens (such as arrestin (S-antigen), recoverin, rhodopsin, melanocyte-associated tyrosinase, interphotoreceptor retinoid-binding proteins, and retinal pigment epithelium-associated antigens) are revealed, which results in a type IV autoimmune response [2]. This phenomenon is usually an effect of ocular trauma but sometimes can be attributed to ophthalmological surgery procedures (especially pars plana vitrectomy [3]), infections and any other causes in which eye tissues are damaged [2,3]. Historically, SO has often resulted in blindness, but currently it can be successfully treated by immunosuppression, usually in the form of corticosteroid administration [3]. Melanoma is the most prevalent intraocular tumor in adults [4]. This tumor arises from melanocytes, usually from the uvea (83% of cases) and is most prevalent in Caucasian populations. The standard treatment procedures include resection, radiation (brachytherapy), and enucleation [4].

In this case report, we would like to present the history of 59-year-old, Caucasian male patient with sympathetic ophthalmia as a consequence of uveal melanoma (UM). 

## 2. Case Report

**Figure 1:**
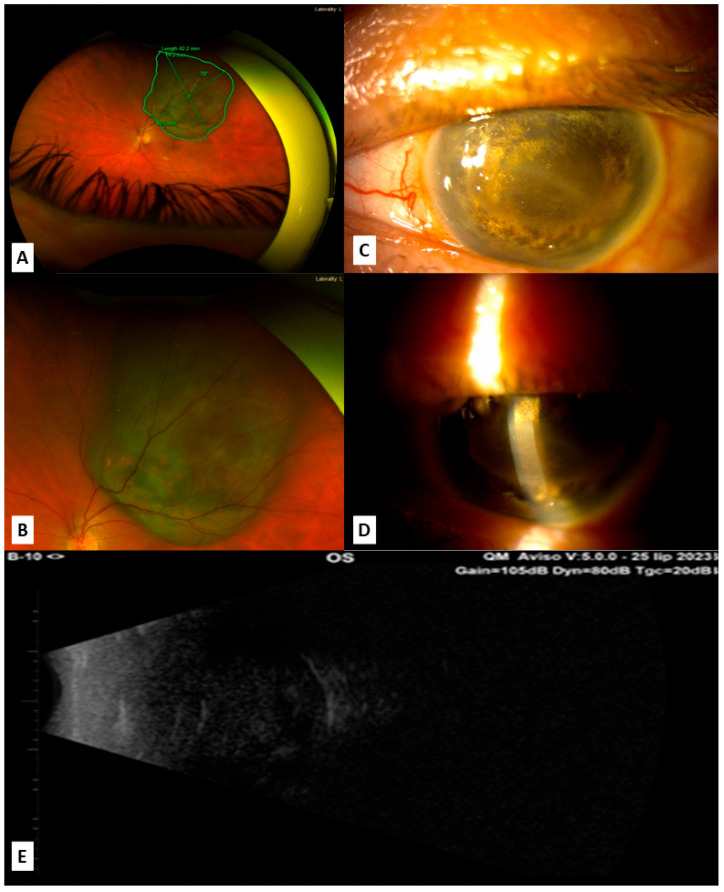
Images showing the left eye of the patient; (**A**) measurement of the melanoma at the moment of first admission; (**B**) melanoma at the moment of first admission; (**C**,**D**) slit lamp examination of the left eye (OS) in the moment of SO appearance in OD, 2 years after initial tumor diagnosis: complete shallowing of the anterior chamber due to tumor masses in the posterior segment of the eye; (**E**) ultrasound at the moment of SO appearance shows that left eye is completely filled with tumor masses.

The patient was referred to the Department of General and Pediatric Ophthalmology at the Medical University of Lublin with a suspected tumor in the left eye. Ophthalmological examination of fundus revealed a choroidal melanoma of the size 14.00 mm × 12.50 mm, with a thickness of <10 mm (Table 1, Figure 1).

Visual acuity (VA) was 0.8 in the right eye (OD) and 0.6 in the left eye (OS). The patient was referred to the ophthalmological oncology center, Suggested option of treatment included brachytherapy and possible need of enucleation. However, the patient declined all proposed treatments.

After 2 years, the patient returned with no light perception in the left eye and significant visual deterioration (counting fingers at a distance of 1 m) in the right eye as well as headaches. The patient reported attending “treatment” for the left eye tumor performed by a person without medical qualifications during the two-year gap from ophthalmologic care. The patient had used unproven “alternative, natural medicine” methods instead of the recommended brachytherapy and/or enucleation—tumor was “treated” with exposure to the magnetic field.

Fundus examination of the right eye revealed a red reflex with no visible details due to vitritis. In USG examination, there was inflammatory exudate and choroidal thickening. The left eye was completely filled with a tumor and showed signs of atrophy (Figure 1).

Suspicion of sympathetic ophthalmia in the right eye was raised, and differential diagnosis included testing for antibodies against Treponema pallidum and Borrelia burgdorferi, which were negative.

**Figure 2:**
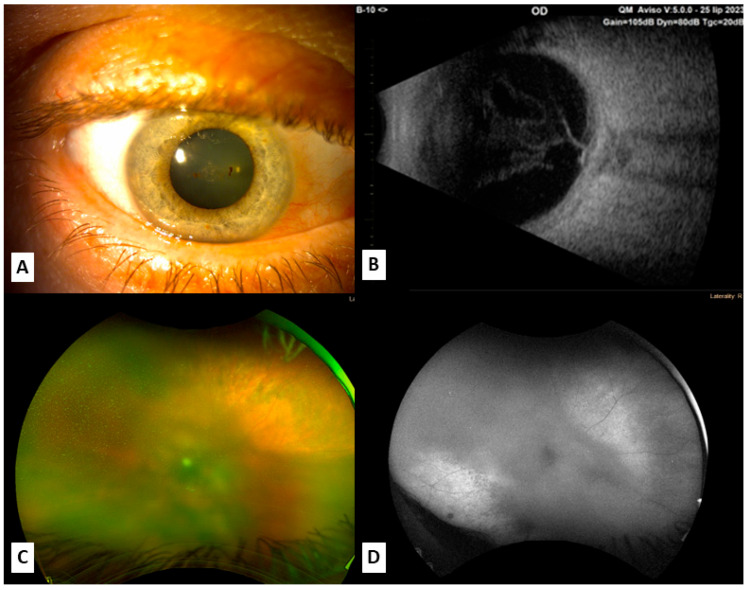
Images showing the right eye; (**A**) slit lamp examination of the right eye at the moment of SO appearance; (**B**) ultrasound at the moment of SO appearance shows vitreous opacities and thicker choroid in the right eye; (**C**,**D**) fundus photo and autofluorescence of the right eye revealing destructive processes of choroid and retina.

The patient was prescribed dexamethasone locally and oral prednisone of 50 mg daily. During hospitalization, an abdominal ultrasound revealed hyperechoic changes in the liver, and subsequent CT scans of the head, chest, abdomen, and pelvis showed no metastases. The liver changes were classified as hemangiomas. When transparency of vitreous increased, fundus photo and autofluorescence were performed, revealing multifocal choroidal lesions, which may most likely correspond to Dalen–Fuchs nodules, although the poor visibility of the interior of the eye prevented a definitive confirmation of the suspected nature of the findings (Figure 2).

For 3 months, sympathetic ophthalmia in the right eye was treated with systemic and topical steroids. Visual acuity in the right eye increased up to 0.3, while the left eye stayed without light perception. The patient developed early cataracts and vitreous opacities.

After 1 year of treatment, visual acuity in the right eye was 0.1 and the left eye was without light perception. During this time, the patient rejected several propositions of standard treatment for a melanoma of this size and agreed only to treatment with steroids. At this time, the patient was treated for sympathetic ophthalmia with prednisone in the dose of 10 mg. The patient was again informed of the need for enucleation of the left eye as his vision had worsened to the verge of blindness, and this time, he consented to the procedure.

The patient was admitted for the planned enucleation of the left eye by standard procedure. At this moment, his visual acuity was as follows: OD counting fingers at 1 m; OS no light perception.

The surgery proceeded as standard, without any additional complications. During the operation, no infiltration of external tissues by the tumor was observed. Following the surgery, an OCULFIT 20 mm implant was used. The patient was in good overall condition post-surgery. Histopathological examination revealed malignant spindle cell melanoma [5] with areas of necrosis and hemorrhage and a sparse inflammatory infiltrate numerous mitotic figures—up to 3 per field, and immunohistochemical readings of HMB45+, S100+, Ki 67 (+)—in about 15% of the tumor cells, and melanA (+).

After enucleation, the patient’s visual acuity in the right eye was counting fingers of the raised hand from 1 m (VA 1/60). The inflammation symptoms were greatly reduced. The patient was informed that the steroid-induced cataract in his right eye could be removed through phacoemulsification to improve his visual acuity.

## 3. Discussion

This rare case illustrates that sympathetic ophthalmia can be caused not only by ocular trauma but also by a neoplastic lesion located in the eye.

Historically, it has been reported that choroidal melanoma can be a cause of SO [6], although in the contemporary literature there has been only one described case (Viswanathan et al., 2018) of SO being an effect of uveal melanoma without other traumatic or surgical causes [6]. In other case reports regarding the coexistence of SO with UM, there was usually a described direct link between SO appearing and previous radiotherapy [7,8,9] or surgical removal of the UM [10]. A literature summary concerning co-existence of SO with UM is displayed in Table 2.

As histopathological examination revealed malignant spindle cell melanoma with areas of necrosis and hemorrhage, it is important to note that such a tumor can expose ocular antigens through mechanisms such as Bruch’s membrane rupture [12,13]. The necrosis found in the tumor can also be linked with this phenomenon, as scleral necrosis in a tumor treated with radiotherapy was found in the literature to initiate SO [9].

SO should be differentiated from other similar non-infectional and infectional causes of uveitis, as was done with our patient for Vogt–Koyanagi–Harada (VKH) syndrome (patient had no systemic symptoms), syphilis (negative serology), borreliosis (negative serology), tuberculosis (no reported contact and no changes in the thorax CT), sarcoidosis (no changes in thorax CT), multifocal choroiditis, and infectious uveitis [3,14,15].

Treatment strategies for SO include immunosuppression and surgical evisceration or enucleation of the excited eye [3]. For uveal melanoma, therapeutic options include radiotherapy and surgical excision [4]. Both options against UM were put forward, but the patient refused them and started seeking “unconventional, natural medicine”. When SO had developed, the patient agreed to immunosuppressing therapy including steroid administration p.o and locally, and finally, when the left eye was filled with the tumor mass, and the right eye was suffering from SO for more than a year, the patient agreed to enucleation.

The demonstrated case not only presents a rare ophthalmic disease but serves also as a cautionary tale. In the case of our patient, a severe complication such as sympathetic ophthalmia could have been avoided if he had undergone the standard therapy [14] proposed at the beginning of treatment. One can also conclude that SO had developed during the time in which the patient was refusing the proposed therapy (brachytherapy or surgical excision of the UM) and instead underwent the “alternative treatment” proposed by a person without professional medical education that had not resulted in any visible improvement in the patients’ condition, and had given the time for SO to develop. Alternative therapy can even be a cause of uveitis [16]—a Chinese herb, Goreisan was reported to cause tubulointerstitial nephritis and uveitis (TINU) syndrome.

The main limitation of our study is the fact that there is no documentation of patient’s medical history during the 2 years in which the patient had refused ophthalmic care and attended only “alternative” treatment that possibly could have influenced the occurrence of SO. For this time period, we rely on the information gathered from the patient, who had admitted to attending “magnetic field exposure” therapy and denied usage of herbal medicines or unprofessional and unauthorized surgical interventions. The other factor severely influencing the outcome of therapy was patient’s refusal to many proposed forms of treatment.

Despite successful treatment and hammering autoimmune inflammatory reaction in the eye affected by SO, the final visual acuity remained low (finger counting from 1 m). This could be explained directly by the destructive processes to the retina and choroid, the formation of an epiretinal membrane, and the side effects of steroid therapy—cataractogenesis.

## 4. Conclusions

This case illustrates that sympathetic ophthalmia can also be an effect of untreated uveal melanoma and not only of its treatment. Early applied brachytherapy or excision of the tumor could have possibly prevented the occurrence of sympathetic ophthalmia in the second eye. The presented case report illustrated rare consequences of delay in the proper treatment of melanoma. In a span of two years, a person with good bilateral vision and a curable disease became practically blind in their only eye. Early applied treatment of uveal melanoma is crucial for the outcome and for lowering the possibility of complications. As demonstrated here, patients’ refusal to proposed treatment as well co-existing medical conditions severely limit the possibilities for treatment, lowering the chance for the preferable outcome. Treatment using only steroids and late enucleation resulted in low final visual acuity in the eye with sympathetic ophthalmia.

## Figures and Tables

**Table 1:** Medical history summary.

Time	Right Eye	Left Eye
29 September 2021	Physiological	Uveal melanoma diagnosis in the left eye and patient’s refusal of treatment
31 March 2023	After 2 years of “alternative therapy” without ophthalmic care, patient has returned with SO in the right eye (VOD = 0.1). Implementation of steroid treatment.	Completely filled with tumor masses. Refusal of treatment.
6 June 2023	Continuation of treatment with steroids (VOD = 0.3), reduction in SO symptoms, incipient post-steroid cataract	Completely filled with tumor masses. Refusal of treatment.
5 June 2024	Symptoms of SO greatly reduced; post-steroid cataract and numerous vitreous floaters (VOD = 0.1)	Completely filled with tumor masses—patient agrees for enucleation of the left eye. Surgery performed on 12 June 2024.
10 July 2024	Post-steroid cataract (VOD = 0.1). Surgical intervention for the cataract was recommended.	Left eye enucleated. Histopathological examination revealed spindle cell melanoma.

**Table 2:** Literature review of sympathethic ophthalmia in choroidal melanoma case reports.

Paper	Melanoma Status	Precipitating Factor for SO	Patient Age	Outcome
Fries PD et al., 1987 [11]	Choroidal melanoma OS	Helium ion irradiation	76	Enucleation of the left eye; BCVA 20/30 OD at SO appearance.
Garcia-Arumi J et al., 2006 [10]	Spindle cell melanoma of the iris and ciliary body OS	Melanoma resection	49	OD: BCVA 20/200 after systemic steroid and immunosuppressive (cyclosporine and azathioprine) treatment of SO, before the surgery for cataract that developed during treatment; OS: BCVA 20/25.
Ahmad N, Soong TK, Salvi S, Rudle PA, Rennie IG., 2007 [8]	Ciliary body malignant melanoma	^106^Ru plaque brachytherapy	41	Vision OU 6/5 after intensive steroid treatment.
Brour J et al., 2012 [7]	Large choroidal juxta-papillary melanoma OS	Proton beam irradiation, then transpupillary thermotherapy	56	SO in right eye 7 years after melanoma irradiation treated with steroids, azathioprin, local steroids, focal laser treatment, and multiple intravitreous anti-VEGF injections. BCVA OD after treatment and cataract surgery was 20/40.
Brour J et al., 2012 [7]	Choroidal melanoma with retinal detachment (OD)	Proton beam irradiation and TTT of the scar, then, after complications (painful neovascular glaucoma), enucleation of the blind right eye	63	One year later SO developed in the left eye, VOS = 20/40. After treatment with intravenous and local steroids with antiglaucomatous eyedrops, VOS has returned to 20/20.
Brour J et al., 2012 [7]	Pigmented supramacular mass of 7.1 mm thickness in OD	Successful treatment with proton beam radiotherapy, complicated with irradiation retinopathy and neovascular glaucoma requiring multiple therapeutic procedures, including panretinal photocoagulation, intravitreal anti-VEGF injection, and cyclocryotherapy	75	After 4 years, SO occurred in the left eye; after treatment with intensive systemic steroids, local steroids, and first-line cyclophosphamide monthly, vision progressed from 20/100 to 20/40 with regression of ocular inflammation and macular edema.
Chen YC et al., 2022 [9]	Primary choroidal melanoma OD	Gamma Knife radiotherapy	55	Five years after radiotherapy SO appeared in the left eye (VOS = 0.7) along with pinkish elevated conjunctival mass in OD. After steroid pulse therapy combined with methotrexate, there was a decrease in the size of the mass in the right eye as well as SO symptoms in the left eye, along with VOS recovery to 6/6.
Viswanathan D et al., 2018 [6]	Six years after SO, choroidal melanoma of the left eye was diagnosed	Ocular trauma 18 months before SO or untreated choroidal melanoma	41	Right eye SO was treated with oral steroids and mycophenolate, cyclosporin, and methotrexate. Despite maximal tolerated treatment, OD developed persistent cystoid macular edema—treated with triamcinolone. After phacoemulsification of steroid-induced cataract, VOD was 20/40, and after 4 years, reduced to hand movement recognition. Six years after initial SO, left eye choroidal melanoma was diagnosed and eye was exenterated.

## Data Availability

The original contributions presented in this study are included in the article. Further inquiries can be directed to the corresponding author.
